# The Frequencies of Immunosuppressive Cells in Adipose Tissue Differ in Human, Non-human Primate, and Mouse Models

**DOI:** 10.3389/fimmu.2019.00117

**Published:** 2019-02-05

**Authors:** Ariane Laparra, Sabine Tricot, Mélanie Le Van, Abderaouf Damouche, Jennifer Gorwood, Bruno Vaslin, Benoit Favier, Stéphane Benoist, Raphael Ho Tsong Fang, Nathalie Bosquet, Roger Le Grand, Catherine Chapon, Olivier Lambotte, Christine Bourgeois

**Affiliations:** ^1^CEA - Université Paris Sud 11 - INSERM U1184, Immunology of Viral Infections and Autoimmune Diseases, IDMIT Department, IBFJ, Fontenay-aux-Roses, France; ^2^Assistance Publique Hôpitaux de Paris, Hôpital Bicêtre, Service de Chirurgie Digestive et Oncologique, Le Kremlin-Bicêtre, France; ^3^Université Paris Sud, Le Kremlin Bicêtre, France; ^4^Assistance Publique-Hôpitaux de Paris, Service de Médecine Interne et Immunologie Clinique, Groupe Hospitalier Universitaire Paris Sud, Hôpital Bicêtre, Le Kremlin-Bicêtre, France

**Keywords:** adipose tissue, Treg-regulatory T cell, immunosuppression/immune modulation, human–animal relationships, healthy adipose tissue, age, metabolism and obesity

## Abstract

Although the metabolic properties of white adipose tissue have been extensively characterized, the tissue's immune properties are now attracting renewed interest. Early experiments in a mouse model suggested that white adipose tissue contains a high density of regulatory T cells (Tregs), and so it was assumed that all adipose tissue has an immunosuppressive profile—even though the investigation was limited to visceral body fat in relatively old male mice. This observation was also corroborated by high frequencies of other cell subsets with immunoregulatory properties, such as anti-inflammatory M2 macrophages, and regulatory B cells. Many studies have since evidenced the persistence of pathogens (trypanosomes, *Mycobacterium tuberculosis*, HIV, etc.) in adipose tissue. However, a recent report identified adipose tissue as a reservoir of memory T cells capable of protecting animals upon rechallenge. The immune potential of lean adipose tissue thus remains to be further investigated. Here, we compared the relative proportions of immune cells (and Tregs in particular) in lean adipose tissue collected from humans, a non-human primate (the cynomolgus macaque), and three mouse models. We demonstrated that the proportion of Foxp3+ Tregs in visceral adipose tissue was low in all models other than the C57Bl/6 mouse. These low values were not linked to correspondingly low proportions of effector cells because T lymphocytes (a main target of Treg suppression) were more frequent in cynomolgus macaques than in C57Bl/6 mice and (to a lesser extent) humans. In contrast, the proportions of macrophages and B cells were lower in cynomolgus macaques than in C57Bl/6 mice. We also observed a higher proportion of CD34+CD45- cells (which predominantly correspond to mesenchymal stem cells) in C57Bl/6 mouse and cynomolgus macaques than in humans and both for subcutaneous and visceral adipose tissues. Lastly, a microscopy analysis confirmed predominant proportion of adipocytes within adipose tissue, and highlighted a marked difference in adipocyte size among the three species studied. In conclusion, our study of lean, middle-aged, male individuals showed that the immune compartment of adipose tissue differed markedly in humans vs. mice, and suggesting the presence of a more inflammatory steady-state profile in humans than mice.

## Introduction

Adipose tissue (AT) is composed of both adipocytes and a stromal vascular fraction (SVF) that contains a highly heterogeneous range of immune cells (1, 2). A growing body of evidence has revealed the close interplay between the immune cells and metabolic cells in AT, which appear to have synergistic roles in the development and control of obesity and in thermostasis (1–3). Adipose tissue also underpins the regenerative functions (4–6) performed by mesenchymal stromal cells [also referred to as adipose stromal cells (ASCs)] and hematopoietic stem cells. Hence, it is now clear that AT has multiple immune and regenerative functions as well as crucial metabolic activities.

Adipose tissue shows a strikingly high level of plasticity when affected by metabolic disorders–either quantitatively (e.g., with the infiltration of pathogenic cells) or qualitatively (e.g., following functional changes in the resident AT cells). With regard to immune functions, most studies of lean, healthy individuals have designated lean fat as an immunosuppressive site (1). Indeed, the vast majority of immune cells in AT appear to exert an immunosuppressive function. This profile favors the AT's metabolic activity (by limiting inflammation and ensuring tissue-remodeling) but might be less beneficial when considering immune responses to infections. The metabolic and immunosuppressive properties of Tregs in AT have been extensively described in mouse models. It has been found that obesity affects both the proportions and transcriptional profiles of AT Tregs (7–12). In clinical settings, the impact of obesity on the proportion of Tregs in human AT has been indirectly assessed by detecting *FOXP3* mRNA; these studies have given more heterogeneous results (13–16). In lean animals, AT contains a low proportion of M1 macrophages and a high proportion of M2 (anti-inflammatory) macrophages (17–21). The accumulation of macrophages and the change in macrophage phenotype are robust markers of obesity in AT, and are observed both in humans and mice (22, 23). Eosinophils (a subset present in lean AT) also exhibit anti-inflammatory properties by favoring the persistence of M2 macrophages and the maturation of adipocytes (24, 25). Studies of other immune cell subsets in AT [such as B cells, natural killer (NK) T cells, γδ-T cells, and innate lymphoid cells (ILCs)] are also now being performed–principally in mouse models. In lean animals, the B cells in AT include a regulatory B fraction (26), whereas obesity is associated with a greater proportion of B cells with a pathogenic profile (12, 27, 28). It has also been shown that the NK T cells in lean AT have immunomodulatory activities, and protect the AT from metabolic disorders (29–32). Natural killer T cells and γδ-T cells reside in the AT of lean individuals, and accumulate when metabolic disorders occur (1, 33, 34). Innate lymphoid cells have been studied in both murine and human ATs (35–37). Type 1 ILCs cells can be triggered by signals induced by metabolic stress and are involved in adipose inflammation, whereas type 2 ILCs appear to provide regulatory signals. Murine and human ASCs also exhibit strong immunosuppressive functions (38, 39). Lastly, the immune activity of adipocytes is also under scrutiny. Adipokine production by adipocytes is clearly associated with the development of an anti- or pro-inflammatory environment in AT (40, 41), as assessed, respectively by the secretion of adiponectin and leptin (41–44). Resolvin and other lipid mediators are also involved in the anti- or pro-inflammatory profile (45–48). Adipocytes also express MHC class II, and may therefore have a key role in immune activation (49–51). If metabolic stress is present, the immune properties of adipocytes also change because the cells upregulate their expression of stress markers and can thus generate pro-inflammatory signals (33).

Based on these observations, one can question the ability of AT immune cells to mount an effective local immune response. Although steady-state immune activity might be controlled by the immunosuppressive environment, AT immune cells might be capable of rapid mobilization once danger signals or pathogen have been detected. This type of plasticity (which has been described for metabolic regulation) might efficiently combine immunomodulation (guaranteeing metabolic homeostasis) and a rapid immune response when pathogens are encountered. Alternatively, the striking persistence of various pathogens (52) [e.g., trypanosomes (53, 54), HIV (55–58), and *M. tuberculosis* (59)] in AT in different species strengthens the hypothesis whereby lasting anti-infectious responses are suppressed in AT. We studied this topic in the context of HIV infection by analyzing the composition of the AT in SIV-infected cynomolgus macaques (55) and then in HIV-infected patients (58). Modest changes in the AT immune compartment were detected: a higher proportion of SVF cells and CD8 T cells, and a modest change in the macrophages' phenotype and T cell activation in SIV-infected animals. In fact, one of our most striking observations was that the basal composition of AT in the cynomolgus macaque and in humans did not fully corroborate the data obtained in mice. We have observed remarkably low frequencies of AT Tregs in lean, non-human primates (NHPs) (55), and non-obese patients (58). More recently, it has also been found that AT is a reservoir for memory T cells capable of protecting the host upon infectious re-challenge after adoptive transfer (60). The objective of the present study was to evaluate the basal immune properties of “healthy” AT as a prerequisite for evaluating AT's anti-infectious responses. To this end, we compared five different experimental models: three murine models (C57Bl/6, the most frequently used model of obesity, CBA and Balb/c strains), the cynomolgus macaque, and healthy human donors. Given that the AT's composition is thought to be strongly influenced by sex, age, and the metabolic context (10, 12), we confirmed these observations in various murine models and then performed comparative analyses of middle-aged male individuals from all species. After using the same protocol for AT dissociation, we performed FACS analyses on the SVF and thus defined the relative contribution of the main immune cell subsets in AT for all the groups tested. We found that a high proportion of AT Tregs was observed in middle-aged male C57Bl/6 mice but not in middle-aged male CBA mice, Balb/c mice, cynomolgus macaques, or humans. Similarly, C57Bl/6 mice had higher proportions of the immune subsets generally associated with immunosuppression (i.e., macrophages, B cells, and γδ-T cells) and ASCs (but not T cells), relative to humans. These discrepancies suggest that the steady-state immunosuppressive environment in AT differs in the three models. Our present observations may have a major impact on the choice of the most appropriate model for studying immune phenomena in the AT.

## Materials and Methods

### Biological Materials

#### Samples From Mice

C57Bl/6 Rj, CBA, and Balb/c mice (Janvier Labs, Saint Berthevin, France), and Foxp3-GFP mice (Charles River, Saint-Germain-sur-l'Arbresle, France) were maintained under pathogen-free conditions in the central animal facility at the Paris-Sud Faculty of Medicine (Paris, France) (approval reference: D92-032-02). All procedures were conducted in compliance with French and European Union regulations on animal welfare (agreement B-94-043-12 and license 94-440, delivered by the French veterinary authorities) and had been validated by the local animal care and use committee (*Comité d'éthique en experimentation animale* (CEEA) 27, Paris-Sud University, Paris, France).

We studied male mice aged 2–3, 6, 12 months, and (for Foxp3-GFP mice only) 18–22 months. The impact of sex on the AT's immune properties was investigated by studying 2–3 months-old male vs. female C57Bl/6 Rj mice. To assess the impact of obesity on the AT's immune properties, 8 week-old C57Bl/6 Rj mice were fed a high fat diet (HFD, Research Diets, Broogarden, Lynge, Denmark), or standard chow for 48 weeks. The animals' bodyweight was recorded weekly. At sacrifice (at 12 months of age), the mean ± standard deviation bodyweight was, respectively 52.0 ± 2.8 and 33.1 ± 3.8 g. Six month-old mice were used in comparisons of C57Bl/6, CBA, and Balb/C animals.

Samples of subcutaneous AT (SCAT) and gonadal AT (as a source of VAT) were collected from all animals. All comparative experiments included appropriate control animals at the time of sacrifice. Different animals were used to study the respective impacts of sex, age, and metabolic disorders. For 2-3 month-old animals, AT samples were collected from two to four mice and pooled in order to obtain sufficient quantities of tissue.

#### Samples From Cynomolgus Macaques

Adult cynomolgus macaques were imported from Mauritius and housed in the animal facility at the *Commissariat à l'Energie Atomique et aux Energies Alternatives* (CEA, Fontenay-aux-Roses, France). The NHPs are housed and handled in accordance with French national regulations, and are subject to inspection by the veterinary authorities (CEA permit number: A 92-032-02). The CEA facility complies with the Standards for Human Care and Use of Laboratory Animals published by the US Office for Laboratory Animal Welfare (reference: OLAW #A5826-01). The use of NHPs at the CEA also complies with the European Directive 2010/63/EU. The animals were used under the supervision of the veterinarians in charge of the animal facility. Animals were housed in adjoining, individual cages (allowing social interactions) and under controlled humidity, temperature and light conditions (12 h light/12 h dark cycles). Water was available *ad libitum*. Animals were monitored and fed (by trained personnel) with commercial macaque chow and fruits once or twice daily. Samples of SCAT and VAT from 10 non-obese, healthy, middle-aged, male cynomolgus macaques were collected at sacrifice. For ethical reasons, the animals were not sacrificed for the sole purpose of collecting AT. Samples from control groups designated in various other studies were made available for the present work. The study protocols were reviewed by the CEA's Animal Care and Handling Committee (*Comité d'Ethique en Expérimentation Animale*, registered with the French Ministry of Research). At the time of the experiments, the median [interquartile range] age was 6.4 years [5.1–7.5], and the median [interquartile range] bodyweight was 7.5 kg [6.3–8.3].

#### Samples From Humans

Subcutaneous AT and/or VAT samples from 10 middle-aged male individuals were collected during elective abdominal surgery. The exclusion criteria were inflammatory bowel diseases, ischemic colitis, cancer, HIV infection, obesity and/or metabolic disorders, weight loss surgery, and the administration of medications with metabolic effects. At the time of surgery, the men's median [interquartile range] age was 53 years (47–59), and the median [interquartile range] BMI was 23 (22–25). Written, informed consent was provided by all participants. The study protocol was approved by the local investigational review board (*Comité de Protection des Personnes Ile-de-France VII*, PP12-021, Paris, France). Samples of abdominal SCAT, VAT, and (when available) blood were collected. We rarely had access to both human SCAT and VAT from the same patient (collection of both SCAT and VAT samples in sufficient quantity was only obtained in 3 out of 10 patients).

### Dissociation of Adipose Tissue

Samples from all species (summarized in Table 1) were processed according to the same protocol. Stromal vascular fraction was isolated from fresh samples. Subcutaneous AT and VAT samples were weighed, washed twice in PBS 1x supplemented with 5% fetal bovine serum (FBS), cut into pieces of 2–3 mm, and then digested in a bath of collagenase (C2139, Sigma-Aldrich, Saint Quentin Fallavier, France) at a concentration of 0.33 mg/mL in DMEM with 5% FBS for 30 min and at 37°C, with continuous shaking. The same batch of collagenase enzyme was used for all species. Next, the tissue was mechanically dissociated by repeated suction into and discharge from a 10 mL syringe. The adipose suspension was then filtered through a 100-micron mesh, and treated with ammonium-chloride-potassium lysing buffer. Cells in the SVF (i.e., all cells other than adipocytes) were treated with Trypan blue (to exclude dead cells) and then counted in Malassez chambers (C-chip, NanoEntek, Seoul, Korea) under the microscope.

**Table 1:** Age, sex, and characteristics of the models studied.

	**Nber**	**Age (median)**	**Sex**	**Metabolic profile**	**Body weight (median)**
**MICE**					
C57Bl/6 Rj	40	2–3 months	M/F	Lean (standard chow)	ND
	10	6 months	M	Lean (standard chow)	25.0 g
	30	12 months	M	Lean (standard chow)	33.1 g
	8	12 months	M	Obese (high-fat diet)	52.0 g
CBA	10	6 months	M	Lean (standard chow)	35.0 g
Balb/C	10	6 months	M	Lean (standard chow)	25.0 g
Foxp3 GFP	12	2–3 months	M	Lean (standard chow)	ND
	6	12 months	M	Lean (standard chow)	ND
	6	18–22 months	M	Lean (standard chow)	ND
**CYNOMOLGUS MACAQUES**					
	10	6.4 years	M	Non-obese	7.5 kg
**HEALTHY HUMAN DONORS**					
	10	53 years	M	Non-obese (BMI 23)	nd

*ND, not determined*.

*Samples of SCAT and VAT were collected from C57Bl/6, CBA and Balb/c mice, cynomolgus macaques, and humans. The quantity of AT obtained differed as a function of the site (SCAT or VAT) and the species. Human SCAT or VAT were predominantly collected from different individuals. In contrast, SCAT and VAT were collected from the same individuals in mice and macaques. If cell numbers recovered from murine samples (especially the younger animals) were not sufficient, samples from 2 to 4 animals were collected for performance of the FACS analysis*.

### Staining for Fluorescence-Activated Cell Sorting (FACS)

The following antibodies were used to stain the cynomolgus macaque and human samples [target molecule (clone)]: CD16 (3G8)/CD90 (5E10)/CD45 NHP (D058-1283)/CD45 Hu (HI30)/CD4 (RPA-T4)/CD8 (RPA-T8)/CD20 (2H7)/CD3 (SP34-2)/TCR GD (B1.1)/NKG2A (Z199)/HLA-DR (G46-6)/CD14 (M5E2)/CD11b (Bear1)/CD34 (563)/CD146 (P1H12)/CD73 (AD2)/CD235a (HI264)/CD31 (WM59)/PD-1 (EH12-2H7)/CD25 (2A3)/Ki67 (B56)/CD39 (eBioA1)/CD127 (MB15-18C9)/Foxp3 236A/E7)/CD1a (SK9)/CD34 (581)/CD123 (7G3)/BDCA2 (AC144)/CD161 (HP-3G10)/CD94 (DX22)/CD117 (104D2)/IL33R/CD69 (FN50)/TCR Vδ2 (B6)/αβTCR (T10B9-1A-31)/αβTCR NHP (R73)/CD45RA (clone 5H9)/CD27 (O323).

The following antibodies were used to stain the murine samples: CD45 (30F11)/F4/80 (BM8)/CD4 (RM4-5)/CD8 (53-6.7)/TCR GD (GL6)/NK1.1 (PK136)/CD11c (HL3)/TCR beta (H57-597)/CD11b (M1/70)/B220 (RA3-6B2)/PD-1 (29F.1A12)/CD106 (429)/CD90 (OX-7)/CD73 (TY/11.8)/CD31 (390)/CD34 (MEC14.7)/CD44 (IM7)/TCR β chain (57-597)/CD127 (A7R34)/CD25 (PC61.5)/CD3 (17A2)/Foxp3 (FJK-16s)/CD69 (H1.2F3)/CD14 (rmC5-3)/CD19 (1D3)/CD11c (HL3)/CD11b (M1/70)/CD25 (PC61.5)/CD161 (PK136)/CD117 (2B8)/IL33R (RMST2-33).

The staining protocol was identical for all three species. Staining was performed after the saturation of Fc receptors by incubation with Fc block reagent (BD) for 30 min at 4°C. Amine-reactive blue dye (LIVE/DEAD^TM^ Fixable, Life Technologies, Carlsbad, CA, USA) was used to assess cell viability. Cells were incubated with monoclonal antibodies for 15 min at 4°C, washed in PBS 1X/10% FBS, and fixed in commercial fixation solution or permeabilized when required [Intracellular Fixation and Permeabilization Buffer Set (eBiosciences/ThermoFisher Scientific, Waltham, MA, USA)]. The FACS data were acquired on an LSR Fortessa system (BD Biosciences, Franklin Lakes, NJ, USA) and analyzed using FlowJo software (Treestar, Ashland, OR, USA).

### Immunohistofluorescence

Adipocyte tissue (volume: 1 cm^3^) was fixed with 4% PFA in phosphate buffer (pH 7) for 6 h, dehydrated overnight in 30% sucrose PBS at 4°C, embedded in optimal cutting temperature compound, and quickly frozen in isopentate with nitrogen liquid. Cryostat sections (thickness: 20 μm) were then prepared, and incubated at 4°C overnight with antibodies against FABP-4, CD45, CD34, and CD4. The anti-FABP-4 antibody was covalently linked to Alexa Fluorochrome AF55 using a microscale labeling kit (Life Technologies) and NHS ester conjugation. Nuclei were stained with 4′,6-diamidino-2-phenylindole (DAPI). An isotype antibody was used as a negative control for each staining. Immunofluorescence images were examined using an SP8 confocal microscope (Leica, Germany).

### Statistical Analysis

The FACS data from mouse-only experiments are quoted as the median ± SD, and the data from all other experiments are quoted as the median [interquartile range]. The microscopy data are quoted as the median ± standard error or the mean (SEM). All statistical analyses were carried out with GraphPad Prism software (version 7.02, GraphPad Software Inc.). A non-parametric Kruskal-Wallis test was used to compare species. For the mouse-only experiments, a paired, non-parametric Wilcoxon test was used to compare SCAT vs. VAT for each species or strain, and an unpaired, non-parametric Mann–Whitney test (^*^p < 0.05, ^**^p < 0.01, ^***^p < 0.001) was used to compare groups. Student's *t*-test was used to compare microscopy data. In graphs, the *p*-value is indicated as follows: ^*^ < 0.05; ^**^ < 0.01, ^***^ < 0.001.

## Results

### The Proportion of Foxp3 Tregs Among CD4 T Cells in Adipose Tissue Is Low in All Models Other Than the Lean Male C57Bl/6 Mouse

**Figure 1:**
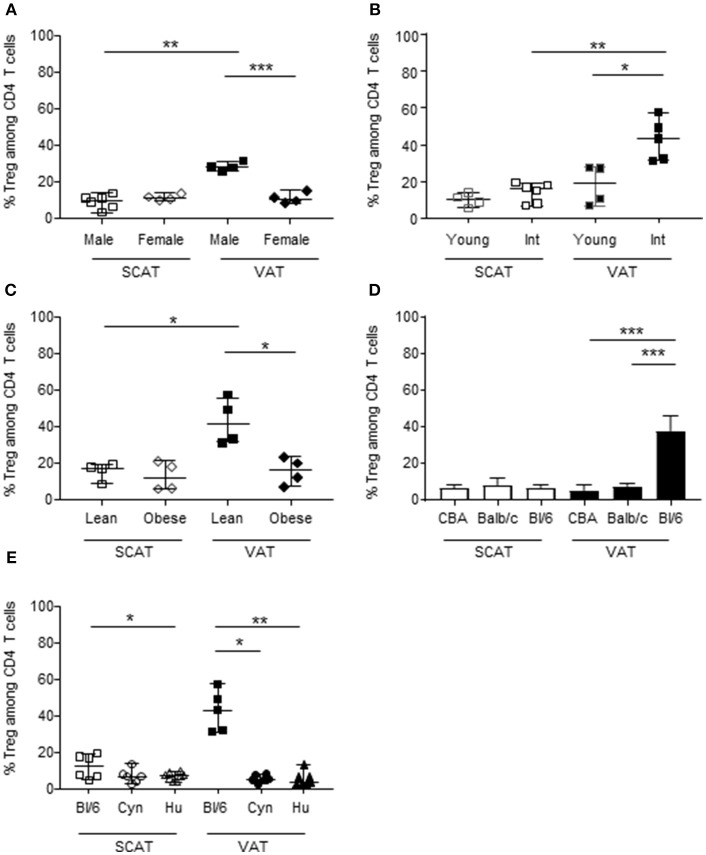
The proportion of Tregs in AT, as a function of sex, age, metabolic context, strain, and species.The frequencies of Foxp3+ cells (calculated as a proportion of CD4 T cells) were quantified in SVF from murine, NHP and human SCAT (open symbols), and VAT (closed symbols). In C57Bl/6 mice **(A–C)**, the frequency of Foxp3+ cells was evaluated as a function of the animals' sex, age, and metabolic context. **(A)** The impact of sex was studied by comparing female with male 8- to 12-week-old C57Bl/6 mice. **(B)** The impact of aging was studied by comparing 8- to 12-week-old vs. 12-month-old male C57Bl/6 mice. **(C)** The impact of the metabolic context was studied by comparing lean vs. obese 12-month-old male C57Bl/6 mice. The data correspond to two representative experiments comparing 4–6 animals. **(D)** Comparison of the frequencies of Foxp3+ cells among CD4 T cells in SCAT and VAT from 6 month-old-male CBA, Balb/C and C57Bl/6 mice. The graphs showed data from 10 mice for each strain. **(E)** Comparison of the frequencies of Foxp3+ cells among CD4 T cells in SCAT and VAT from adult male C57Bl/6 mice (age: 10–14 months), cynomolgus macaques (age: 5–7 years), and humans (age: 47–59 years). Bars represent median values. Statistical significance was determined with a Kruskal–Wallis test (^*^p < 0.05, ^**^p < 0.01, ^***^p < 0.001) when performing comparisons of SCAT vs. VAT within a group, and a non-parametric Wilcoxon test (^*^p < 0.05, ^**^p < 0.01, ^***^p < 0.001) when comparing groups.

We and others have previously demonstrated that Foxp3+ CD4 T cells are extremely scarce in VAT from humans and cynomolgus macaques (55, 58, 61), when compared with the corresponding data obtained in mice (8, 11, 62). To fully ascertain the potential differences in immune cell composition between humans and the other animal models, we analyzed SCAT and VAT samples from three mouse strains, a non-human primate (the cynomolgus macaque) and humans. The method used to dissociate the AT (i.e. the type, batch and concentration of collagenase, and the duration of incubation) was the same for all species. Likewise, the criterion used to identify Tregs (Foxp3 expression by CD4 T cells) was the same for all species. Given that the proportion of Tregs has been shown to differ markedly as a function of sex, age and metabolic context (e.g., obese vs. lean mice) (8, 10), we chose to study mouse models in which all these conditions can be easily evaluated. We first compared the frequency of Foxp3+ CD4 T cells in 2- to 3-month-old male and female C57Bl/6 mice (Figure 1A). The Treg frequency was significantly higher in the VAT of male animals, whereas all other samples (female SCAT and VAT, male SCAT) exhibited values that were similar to those usually reported for the murine spleen. We next studied the impact of age on male C57Bl/6 mice by comparing 2- to 3-month-old and 11- to 13-month-old animals (Figure 1B). In line with the literature data, we confirmed the drastic impact of age on the percentage of Foxp3+ cells in the VAT of male C57Bl/6 mice. We also used Foxp3-GFP C57Bl/6 mice to evaluate the long-term persistence of Tregs in VAT (Supplementary Figure 1). As observed in B6 mice, the percentages of GFP+/Foxp3+ cells in both VAT and SCAT were significantly higher in older animals (12 months of age and 18–22 months of age) than in young animals (i.e., 2–3 months of age); the difference was less striking for SCAT, however. Lastly, as a control, we also evaluated the impact of a metabolic disorder on the Treg frequency in AT by comparing HFD-induced obese animals with lean animals. 8 week-old mice were fed a HFD or standard chow, and sacrificed at 10–12 months of age. As described previously (11, 12), obese male animals had a significantly lower proportion of Tregs in the VAT than their lean counterparts (Figure 1C). Taken as a whole, our results confirmed the literature reports of a high frequency of Tregs in the AT in a very restricted context (i.e., in relatively old, non-obese, male animals) and in a relatively specific tissue (i.e., VAT, rather than SCAT). Given that these observations did not match those reported for cynomolgus macaque and human VAT samples, we next evaluated the proportion of Tregs in the SCAT and VAT of 6 month-old C57Bl/6, CBA and Balb/c male mice. Interestingly, the proportions of Tregs in the SCAT and VAT were much lower in both CBA and Balb/c mice (Figure 1D). We next assessed the proportions of Foxp3+ CD4 T cells in AT samples collected from mice, NHPs, and humans. On the basis of our initial set of experiments, we selected individuals with the following characteristics: male gender, middle age (12 months of age for mice, 6 years of age for cynomolgus macaques, and 53 years of age for humans) and no obesity (mice fed standard chow, a median bodyweight of 7.5 kg for cynomolgus macaques, and a median BMI of 23 for humans). The C57Bl/6 mouse [the best-characterized murine model in studies of obesity and AT homeostasis (11, 63)] was used as control for this comparison. The high proportion of Tregs observed in the VAT of C57Bl/6 mice was not observed in the VAT or SCAT of cynomolgus macaques or humans (Figure 1E).

Overall, we found that C57Bl/6 mouse appears to be a rather peculiar model with regard to the high proportion of Foxp3+ CD4 T cells in the VAT. This high proportion was not observed in two other strains of mice (CBA or Balb/c) or in two other species (the cynomolgus macaque and the human).

### The Low Proportion of Foxp3 Tregs in Cynomolgus Macaques and Humans Impacts the Effector/Treg Ratio in Adipose Tissue

**Figure 2:**
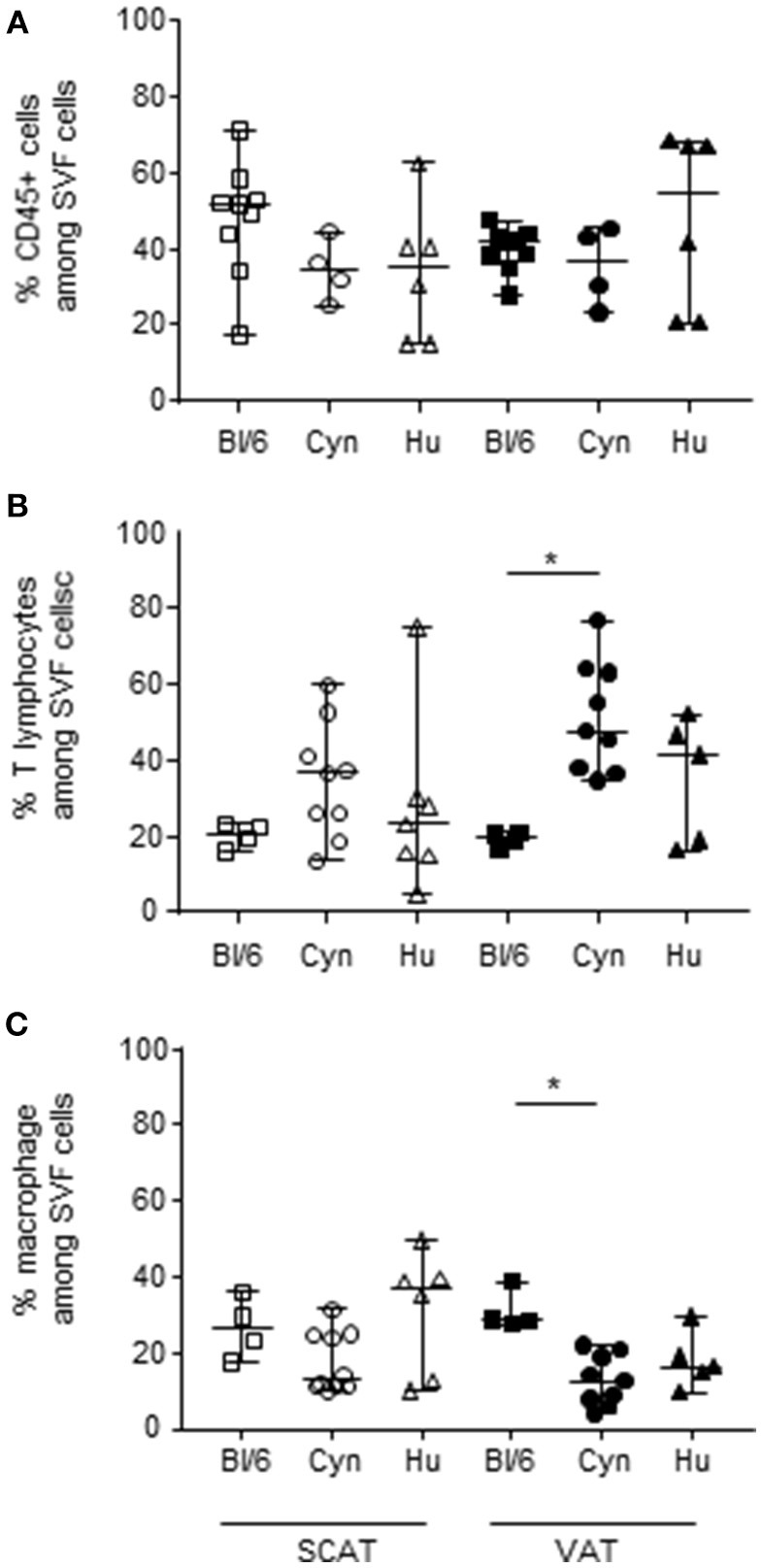
Frequencies of hematopoietic CD45+ cells, macrophages, and T lymphocytes in SVF. The frequencies of CD45+ cells **(A)**, T cells **(B)**, and macrophages **(C)** in the SVF are shown. The comparison was performed on samples of SCAT (open symbols) and VAT (closed symbols) collected from 4 to 10 mice or groups of mice, 10 cynomolgus macaques, and 9 human donors. Macrophage subsets were defined as F4/80+ cells in mice, and CD14+ cells in cynomolgus macaques and humans. (In humans and cynomolgus macaques, HLA-DR and CD11b expression were confirmed on CD14+ cells). T cells were defined as CD3- and/or TCRβ-expressing cells. Bars represent median values. Statistical significance (^*^p < 0.05 in a non-parametric Wilcoxon test) is shown as appropriate.

The immunosuppressive activity of Tregs is also defined by the ratio between the Tregs and the potentially suppressed effector cells. One could therefore hypothesize that the low proportion of Tregs in human and cynomolgus macaques AT (relative to C57Bl/6 mice) essentially reflects the low proportion of effector T cells without affecting the Treg/effector ratio and the AT's immunosuppressive potential. We therefore looked at whether the low proportion of Foxp3 Tregs reflected a difference (i.e., a relative reduction) in the proportion of effector cells in cynomolgus macaques and humans. We first determined the proportions of hematopoietic CD45+ cells (and macrophages and T cells, the two most represented subsets among CD45+ cells) in the SCAT and VAT of the three groups. The three species did not differ with regard to the proportion of hematopoietic cells (Figure 2A). In each species, the proportion of hematopoietic cells was similar in SCAT and VAT. The data were more dispersed within the group of humans, which presumably reflects greater genetic heterogeneity. We next evaluated the proportion of T lymphocytes in SVF cells–a subset generally targeted by Tregs (64). The proportion of T cells in VAT was higher in cynomolgus macaques than in C57Bl/6 mice (Figure 2B). A similar trend was observed when comparing humans with mice. The low proportion of Tregs was thus not attributable to the low proportion of effector T cells. Lastly, we evaluated the proportion of macrophages, which can potentially be modulated by Tregs (65–68) but may also favor Treg accumulation (69, 70) The proportion of macrophages in the SVF fraction was not defined in the same way in all the three species: F4/80 expression in mice, and CD14 expression in cynomolgus macaques and humans. The percentage of monocytes/macrophages in middle-aged male cynomolgus macaques was significantly lower than the percentage of macrophages in C57Bl/6 mice (Figure 2C). A similar difference was observed when comparing human and mice VAT samples, although it was not statistically significant. Although the detection of macrophages may be less stringent in cynomolgus macaques and humans, the proportion in these species was significantly lower than in C57Bl/6 mice. The low proportion of macrophages may indirectly reflect the elevated proportions of T cells observed in humans and macaques, relative to C57Bl/6 mice.

In conclusion, 12 month-old male C57Bl/6 mice differed from middle-aged male cynomolgus macaques and humans with regard to the macrophage and T lymphocyte profiles in VAT. The VAT in C57Bl/6 mice featured a higher proportion of macrophages, whereas the VAT in cynomolgus macaques and humans featured a higher proportion of T lymphocytes. When comparing Treg and effector cell fractions in the three species, we observed that the low number of Tregs (as a proportion of all CD4 T cells) in SCAT and VAT from cynomolgus macaques and humans was associated with a higher proportion of T cells (the usual target of Treg suppression).

### The Relative Contributions of Immune Subsets Among CD45+ Cells in VAT From Middle-Aged Male C57Bl/6 Mice, Cynomolgus Macaques, and Humans.

**Figure 3:**
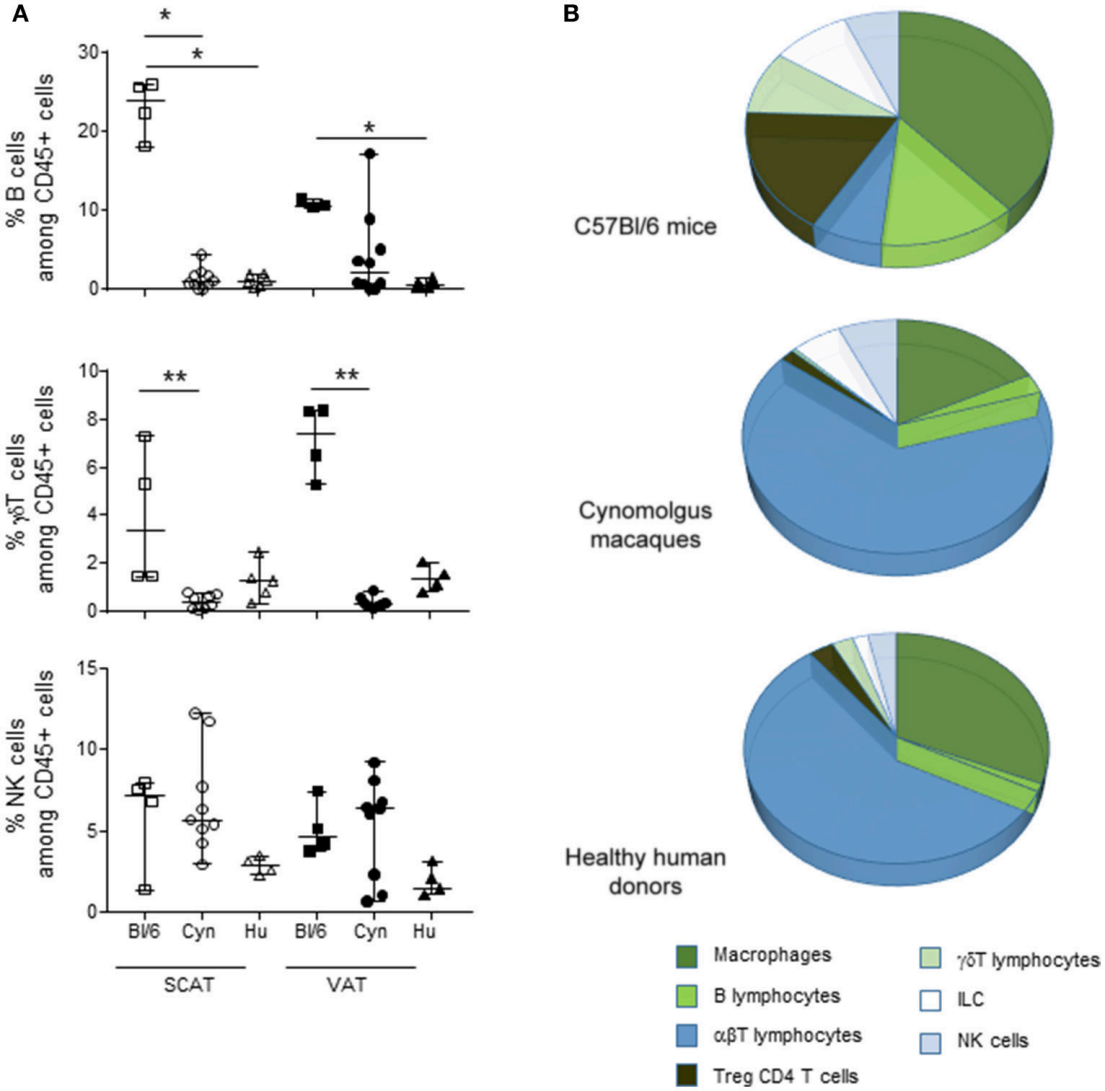
Frequencies of immune cell subsets among the hematopoietic CD45+ SVF cells in VAT. The cell subsets in SCAT and VAT SVF in adult, non-obese, male C57Bl/6 mice, cynomolgus macaques, and healthy human donors were studied using flow cytometry. When considering CD45+ cells, we identified macrophages, B lymphocytes, αβ T lymphocytes, Foxp3 + CD4 Tregs, γδ T cells, ILCs, and NK cells. **(A)** Comparison of the percentages of B cell, γδ T cells, and NK cells among CD45+ cells. **(B)** Pie chart of the cell subsets composing the CD45+ fraction in the SVF for each species. The comparison was performed on VAT collected from 4 groups of mice, 10 cynomolgus macaques, and 9 healthy human donors. Statistical significance (^*^p < 0.05, ^**^p < 0.01, in a non-parametric Wilcoxon test) is shown as appropriate.

Given the observed intergroup differences in the proportions of Tregs, macrophages and T cells, we next performed an overall analysis of the main immune subsets commonly studied in AT. In addition to macrophages and αβ T cells, we determined the relative proportions of B cells, γδ T cells, NK cells, and ILCs (Figure 3A). The identification criteria for each subset in each species are given in Supplementary Table 1. In both SCAT and VAT, the frequency of B cells (as a proportion of CD45+ cells) was markedly higher in C57Bl/6 mice than in humans. A similar trend was observed when comparing C57Bl/6 mice and cynomolgus macaques. In both SCAT and VAT, the percentage of γδ T cells was also higher in middle-aged male C57Bl/6 mice than in cynomolgus macaques. Again, a similar trend was observed in humans. The proportions of NK cells and the ILCs (data not shown) were similar in all groups considered. The relative proportions of the various CD45+ immune cells in the SVF from VAT are shown in Figure 3B. Arbitrary colors were given to each subset, based on the immune function described by the data literature in mice, i.e., an immunosuppressive function (in green) or an effector function (in blue). Macrophages, B cells, γδ T cells, and NK cells have been linked to modulatory properties in lean AT (1, 26), and so were assigned a green color in Figure 3B. Although the functional activity of the various immune subsets was not evaluated in the present study, the relative proportions suggested that the immune cells' environment differ in middle-aged, male C57Bl/6 mice vs. humans, and cynomolgus macaques.

### The High Proportion of CD34+CD45- Cells in SVF Collected From Middle-Aged Male C57Bl/6 Mice Was Not Observed in NHPs and Humans

**Figure 4:**
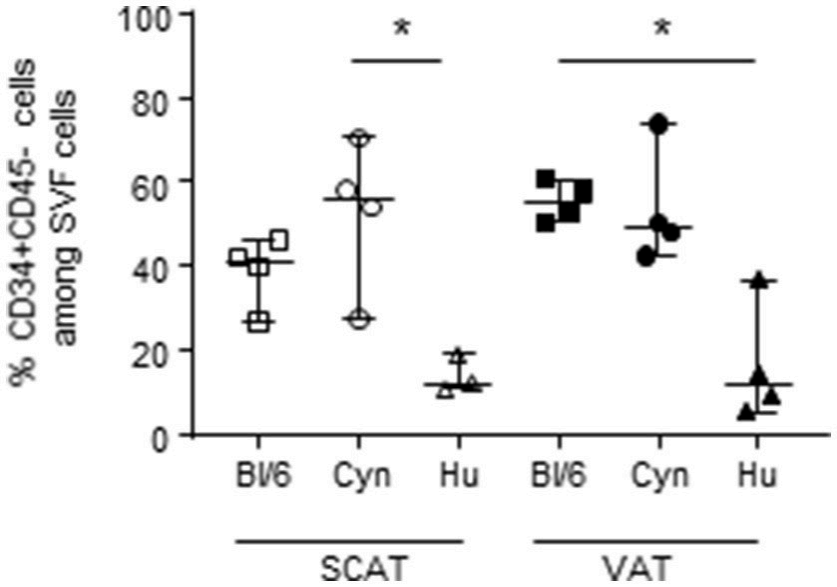
Frequencies of CD34+ CD45- cells in SVF. The frequency of CD34+CD45- cells in the SVF from SCAT and VAT in adult, non-obese, male C57Bl/6 mice, cynomolgus macaques, and healthy human donors. We assessed SCAT and VAT collected from 4 groups of mice, 4 cynomolgus macaques, and 5 human donors. Statistical significance was determined with a Kruskal Wallis test when performing comparisons of SCAT vs. VAT within a group, and a non-parametric Wilcoxon test (^*^p < 0.05) when comparing groups.

To provide an exhaustive characterization of the potential immunosuppressive environment in AT, we also evaluated the percentage of CD34+CD45- cells in the SVF of the three species considered. The CD34+CD45- fraction is enriched in AT mesenchymal stromal cells (i.e., ASCs). These cells are multipotent progenitors that have attracted great interest in the context of regenerative therapies based on AT extracts. However, ASCs are also characterized by their high immunosuppressive potential. Although the ASCs' immunosuppressive mechanism has not been fully characterized, these cells express CD73—an ectonucleosidase that triggers immunosuppressive pathways. We thus evaluated the proportion of CD34+CD45- in the SVF from SCAT and VAT of the three groups studied (Figure 4). Interestingly, the percentages of CD34+CD45- cells in SCAT and VAT (ranging from 40 to 60%) were similar in middle-aged male C57Bl/6 mice and cynomolgus macaques but were significantly lower in middle-aged male humans. We also determined CD73 expression by CD34+CD45- cells in the SVF in cynomolgus macaques and humans. As previously described in mice, we observed that most of the CD34+CD45- cells expressed CD73 (data not shown). Our analysis of the CD34+CD45- fraction identified a high proportion of non-hematopoietic cells with potential immunosuppressive properties in C57BL/6 mice and cynomolgus macaques but not in humans.

### Adipocyte Size Also Differed When Comparing Middle-Aged Male C57Bl/6 Mice, Cynomolgus Macaques, and Humans.

**Figure 5:**
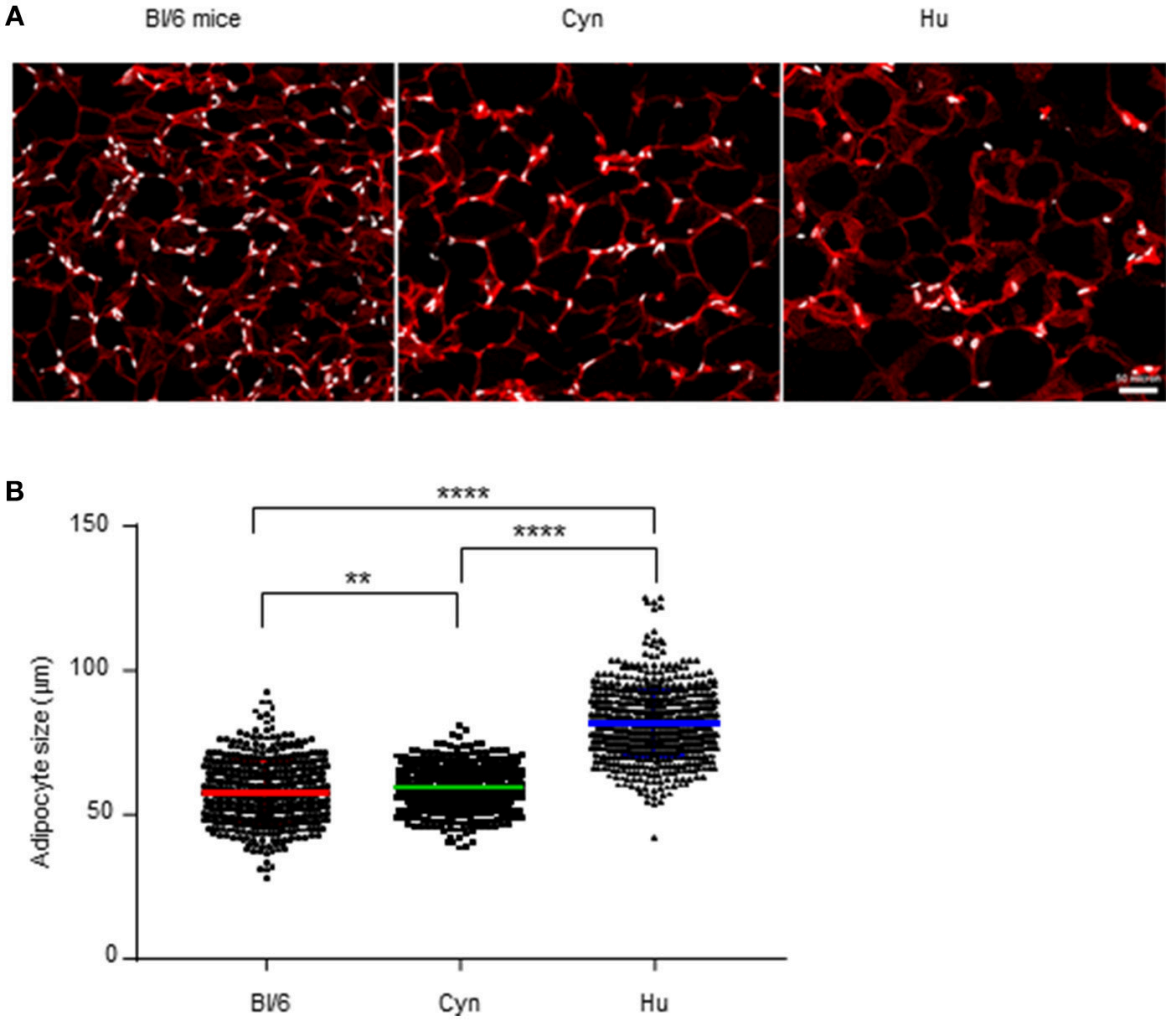
Relative distributions of adipocytes and SVF cells. **(A)** Immunohistofluorescence image of adipocytes (FABP-4 staining, in red) and SVF nuclei (DAPI staining, in white) from VAT collected from non-obese male C57Bl/6 mice, cynomolgus macaques, and healthy human donors. **(B)** We measured the adipocyte size (using Image J software) on 5 images for each sample. Each point represents the size of one adipocyte. The mean ± SEM and the 95% confidence interval are shown. The graph represents the pool of the 3 samples tested for each specie. Statistical significance was determined in an unpaired *T*-test (^**^p < 0.01, ^****^p < 0.0001).

To more accurately evaluate the immune environment within AT, we performed an immunohistofluorescence analysis of VAT tissue sections. In fact, flow cytometry analysis of the SVF requires prior tissue dissociation and thus eliminates the adipocyte fraction–the main cell type in AT. Our immunohistofluorescence analyses detected FABP-4-expressing adipocytes in VAT collected from lean, middle-aged, male C57BL/6 mice, cynomolgus macaques, and humans (Figure 5A) but also simultaneously detected CD45-expressing cells and CD34-expressing cells. We found that the proportions of CD45- and CD34-expressing cells in all three groups were extremely low. Indeed, these cells were absent in a large number of fields (data not shown). Although some aggregates were observed, the size of the overall immune compartment in AT samples in this study of male, middle-aged animals, and humans appeared to be extremely small. This observation emphasized that the adipocyte is the key cell in AT in lean healthy individuals, and that AT immune cells are scarce. The flow cytometry strategy used here to phenotype immune cells within the SVF after AT dissociation might overestimate the relative proportions of these cells in AT. The results of tissue section imaging suggested that direct interaction between immune cells and adipocytes probably predominates, given the high proportion of adipocytes.

Importantly, our immunohistofluorescence analyses provided an indirect estimate of the size of the adipocytes in VAT from all groups studied (Figure 5B). The mean ± SD adipocyte size differed significantly when comparing middle-aged male C57Bl/6 mice (57.4 ± 0.5 μm), cynomolgus macaques (59.0 ± 0.2 μm), and humans (81.0 ± 0.4 μm). These results show that the species' ATs differ with regard to both the SVF and the adipocyte compartment.

## Discussion

White AT is a complex tissue with regard to its (i) many metabolic, regenerative and immune functions, (ii) complex cellular interactions, and (iii) high plasticity (as evidenced by the metabolic and immune changes induced by metabolic insults). The renewed interest in the biology of AT has prompted fundamental studies in mice, clinical research in patients, and (in certain infectious contexts) studies of NHPs. However, the steady-state immune properties in each model have not been clearly established, and a review of the literature data suggests that there are major discrepancies between the models in this respect. To fully appraise the immune properties of AT in the respective models, we compared “healthy,” middle-aged male rodents, NHPs and humans. Our results highlighted a number of differences in the AT between the various models. Before addressing the composition of the stromal vascular fraction of adipose tissue, we first aimed to analyze the density of SVF cells per gram of AT in the various models by counting SVF cells recovered following collagenase treatment. Although various exclusion criteria were used for the selection of “healthy” subjects, we still observed important heterogeneity in SVF cell recovery, precluding any definitive conclusion to be drawn between the three models. We then aimed to evaluate the density of CD45 expressing cells by immunohistofluorescence (that allowed to provide co-staining for adipocytes (FABP-4), CD45, or CD34 expressing cells). A potential bias is the heterogeneity in SVF cell density as a function of the location within AT (perivascular vs. deeper area of AT). Analyses (performed here on deep sections of AT) showed a limited density of SVF CD45+ cells, precluding any comparison. Lastly, microscopy analyses showed differences in adipocyte size and density as a function of the model. We were thus not even convinced that the comparison of SVF cell numbers per gram of AT would be appropriate. Due to these technical hurdles (heterogeneity, limited density, change in adipocyte size as a function of the model), we did not introduce any data on the number of SVF cells collected per gram of AT in the current manuscript. However, the comparison of the composition of AT in the various model is robust. A striking observation was that the Treg frequency was much higher in the C57Bl/6 model than in the other mouse strains or in primates. Based on the proportions of many immunosuppressive subsets, the AT's immunomodulatory profile appeared to be more “potent” in the C57Bl/6 model than in primates. The proportions of macrophages and ASCs were lower in human VAT than in murine VAT, whereas the overall proportion of T cells was higher. The differences in B cells, γδT cells, and ILCs are also suggestive of lower immunosuppressive potential in human AT.

These comparisons are obviously subject to debate. The criterion for “middle age” corresponded to 12 months of age in mice, 6 years of age in cynomolgus macaques, and 53 years of age in humans. Although this comparison appears to be reasonable, it still constitutes a study limitation. The same criticism can be raised with regard to the apparently “healthy” status of all the individuals. Although healthy mice and cynomolgus macaques can be maintained with relative ease, it is more difficult to consider that humans undergoing elective surgery are indeed “healthy.” In the present study, and on the basis of our previous experiments (58), we selected male patients undergoing cholecystectomy because the latter condition is usually not related to immune or metabolic disorders. The exclusion criteria included the presence of inflammatory bowel diseases, ischemic colitis, cancer, HIV infection, obesity, metabolic disorders (such as diabetes), and the administration of medications with metabolic effects. Despite the application of these precautions, we nonetheless observed major discrepancies between the models. Lastly, one must also take into account the heterogeneity of the AT's immune composition as a function of its location in the body. Although we analyzed both SCAT and VAT, the latter corresponded to gonadal fat in mice and abdominal omental fat in cynomolgus macaques and humans. Although gonadal fat is widely considered to be a robust model of VAT (71) (notably due to the limited quantity of abdominal VAT in mice), this difference must be taken into account. In this respect, a comparison of the three murine strains was crucial because the CBA and Balb/c mice exhibited much the same profile as the NHP and humans but differed from C57Bl/6 mice—suggesting that the intrinsic properties of gonadal AT are not responsible for the observed differences.

The observed differences between C57Bl/6 mice on one hand and the NHP and humans on the other raise the question of which mechanisms might be responsible. Firstly, one could hypothesize that environmental factors (e.g., differences in the microbiota, the history of infection or even exposure to pollutants) have a role. Interestingly, the observed differences between the mouse strains suggest that a history of infection (which was limited in all three strains, given their housing conditions) does not drive the accumulation of Tregs in AT. However, this strain-dependent heterogeneity may reflect differences in both the microbiota (72, 73) and intrinsic metabolic factors. C57Bl/6 mice exhibit lower gut microbiota diversity, richness and Firmicutes/Bacteroidetes (F/B) ratio compared to Balb/C mice (73). These observation led to various points of discussion. Higher diversity and richness is assumed to be beneficial for host health (74) and may thus protect Balb/c animals from metabolic disorders and obesity. The F/B ratio is also considered a significant marker of gut microbiota composition and high F/B ratio is associated with obesity (75, 76). Although a more refined characterization of the bacteria of both phyla is required, this variation in the composition of microbiota may provide an important rationale for the different susceptibility of the mouse strain to obesity and/or adipose tissue cell composition. Composition of the microbiota has been shown to modulate Treg frequencies in healthy individuals (77, 78) although these studies did not analyze adipose tissue. Altogether, the variation in gut microbiota modulate the development of immune responses and may contribute to the higher sensitivity of the C57Bl/6 strain to obesity (79). It is obviously more difficult to determine which factors are responsible for differences in the baseline composition of “healthy” AT as a function of species. Regardless of the mechanisms involved, our present data suggest that the AT environment comprises less immunosuppressive cells in humans than in the C57Bl/6 reference murine model. However, the lack of functional assay data means that these results should be interpreted with a degree of caution. One can postulate that the immunosuppressive and/or effector properties displayed by a defined cell subset in mice may not be recapitulated in NHP and humans. Alternately, the immunosuppressive properties may be carried on by different cell subsets in the various species. The difference in the frequencies of immunosuppressive cells will have to be taken into account for the development of suppressive assay that may require different stimulation to trigger the immunosuppressive function of the AT immunosuppressive cells. We were thus currently unable to provide a direct functional comparison of the immunosuppressive properties of AT between all species.

However, the persistence of multiple pathogens (HIV (55, 56, 80), *M. tuberculosis* (81), and trypanosomes (82) in human AT indirectly suggests that strong immunomodulation enables the persistence of pathogens in this tissue. It is tempting to speculate that immunosuppressive properties still develop in human AT but rely on mechanisms other than Tregs or other immunosuppressive cells. In a previous report, we described the high proportion of PD-1-expressing (and thus potentially exhausted) T cells in human AT (58) which may contribute to the low efficacy of immune responses. Additionally, our present study of fixed AT sections served as a useful reminder that the most prominent cell in AT (i.e., the main contributor to the AT's specific microenvironment) is the adipocyte—the role of which as an immune regulator remains to be characterized in detail. Indeed, the flow cytometry analysis performed after collagenase digestion induces bias in favor of the SVF at the expense of the adipocytes. Our immunohistochemical results emphasized the very low number of CD45+ cells detected per field, and suggest the adipocytes' predominant role as immune regulators within AT. This observation underlines the importance of studying direct interactions between adipocytes and immune cells (83). A second important observation relates to the difference in adipocyte size in the different models—suggesting that the adipocytes have different metabolic profile. It remains to be seen whether these metabolic properties of adipocytes influence the infiltrating immune cells. Finally, these differences in adipocyte size and density also suggest that transcriptomic analyses may be difficult to analyze due to the differences in adipocyte density either between species or between metabolic contexts. Evaluation of cytokine production in AT by SVF cells may be highly affected by the inflammatory status, but masked or minored by AT associated hyperplasia.

Lastly, our observations raise the question of which animal model should be used to study AT. Given that it is not easy to collect large quantities of AT from healthy subjects, the collection of AT from animal models is still an important means of evaluating this tissue's functional properties. Depending on the research question, each model has specific advantages: the mouse model provide access to various knock-out or knock-in system that might be useful for characterizing specific and possibly complex interactions between cell partners in AT. The choice of the B6 background may enable a more specific focus on the Treg compartment and the mechanisms that might modulate and/or preserve the compartment's activity. The striking differences in Treg proportions in the various mouse strains also questioned the physiological role of Tregs accumulation in AT on metabolic homeostasis and susceptibility to obesity. Considering the regenerative potential of AT, the proportion of ASCs differs from one model to another; the low proportion of ASCs in human samples suggest that the mouse or cynomolgus macaque model is more appropriate for studying these cells. However, the ASCs' functional properties need to be properly assessed in each model. The difference in the proportion of ASCs between humans (low) and the mouse and cynomolgus macaque models (high) was a rare example of a discrepancy between cynomolgus macaques and humans. This finding might indicate that ASCs are highly sensitive to environmental pollutants; whereas murine and cynomolgus macaque models are fairly well-protected from environmental pollutants, healthy humans are obviously exposed to various toxics that can accumulate in AT (84, 85). This hypothesis warrants further investigation. The influence of sex on ASCs should also be investigated further. Indeed, ASCs have been used to treat women (but not men) for systemic sclerosis in clinical trials (86)–suggesting that women have larger numbers of ASCs.

In summary, we compared the cell composition of AT in various experimental models (primarily the C57Bl/6 mouse, the cynomolgus macaque NHP, and humans). We observed marked differences in the proportions of immunosuppressive subset profiles (e.g., Tregs) in these models. Although the mechanisms responsible for this discrepancy are unclear, it is clear that the steady-state immune compartment in AT differed markedly in middle-aged male humans vs. mice. This observation has several implications: (i) the steady-state immune composition of human AT may have to be reassessed by considering age- and sex-matched healthy controls; (ii) the high incidence of obesity in humans may be due to the lower immunosuppressive activity of AT, which therefore protects less well against metabolic insults; and (iii) the adipocyte has a central role in the AT's functions, and there is a low degree of interaction between immune cells in non-obese male human AT.

## Author Contributions

AL, MLV, AD, and JG performed AT dissociation and FACS staining experiments. ST performed the immunohistofluorescence analysis. AL, OL, and SB ensured access to human samples. RH, NB, and RL ensured access to cynomolgus macaques samples. CC, BF, BV, RL, OL, and CB designed the experiments and supervised the analyses. AL and CB wrote the paper. OL and CB supervised the project.

### Conflict of Interest Statement

The authors declare that the research was conducted in the absence of any commercial or financial relationships that could be construed as a potential conflict of interest.

## Supplementary Material

Supplementary Figure 1 The proportion of Tregs in AT, as a function of the age of Foxp3-GFP reporter mice. The frequencies of Foxp3+ cells among CD4 T cells in SVF from SCAT (open symbols) and VAT (closed symbols) in male Foxp3-GFP mice aged 2–3, 12, and 18–22 months. Bars represent median values. Statistical significance was determined in a non-parametric Wilcoxon test (^*^*p* < 0.05).

Supplementary Table 1 Identification strategy for each subsets in the different species.
