# Supplementary material for: The Frequencies of Immunosuppressive Cells in Adipose Tissue Differ in Human, Non-human Primate, and Mouse Models
Source: Front Immunol. 2019 Feb 5;10:117. doi: 10.3389/fimmu.2019.00117 (PMC6371887; doi:10.3389/fimmu.2019.00117)
Supplement: Supplementary Table 1 — Identification strategy for each subsets in the different species. [file Table_1.docx]

| **Subsets** |  | **FACS characterization strategy** | | |
| --- | --- | --- | --- | --- |
|  |  | Mouse | NHP/ Human | |
|  |  |  |  |  |
| CD4+ Foxp3+ Treg |  | CD45+ L/D- CD3+ CD14- CD4+ Foxp3+ | | |
| T lymphocytes |  | CD45+ L/D- TCR βchain+ CD14- | | |
| Macrophages |  | CD45+ L/D-  **F4/80+** | CD45+ L/D-  **CD14+ (CD11b+HLA-DR+)** | |
| B cells |  | CD45+ L/D- CD3- F4/80- **B220+** | CD45+ L/D- CD3- CD11b-/HLA-DR-  **CD19+** | |
| NK cells |  | CD45+ L/D- CD3- CD14-  **NK+** | CD45+ L/D- CD3- CD14-  **NKG2A+** | |
| γδ T cells |  | CD45+ L/D- CD3+ CD14-  TCR βchain- γδ TCR+ | CD45+ L/D- CD3+ CD14-  αβTCR- γδ TCR+ | |
| ILC |  | CD45+ L/D-  **Lin-** (CD14, F4/80, CD19, CD11c, CD11b, FCγRε)  TCR βchain- γδ TCR-  CD25, CD127, NK | CD45+ L/D-  **Lin-** (CD1a, CD16, CD14, CD20, CD123, FCγRε)  TCR βchain- γδ TCR-  BDCA2-  CD25, CD127 | |
| ASC |  | CD45- CD34+ CD31- (CD73+/-) | | |

Supplemental Table 1: Identification strategy for each subsets in the different species
